# Chronic Pain: Structural and Functional Changes in Brain Structures and Associated Negative Affective States

**DOI:** 10.3390/ijms20133130

**Published:** 2019-06-26

**Authors:** Seoyon Yang, Min Cheol Chang

**Affiliations:** 1 Department of Rehabilitation Medicine, Ewha Woman’s University Seoul Hospital, School of Medicine, Ewha Woman’s University, Seoul 07804, Korea; 2 Department of Rehabilitation Medicine, College of Medicine, Yeungnam University, Daegu 42415, Korea

**Keywords:** chronic pain, central sensitization, negative affective state, corticolimbic system, neurotransmitter

## Abstract

Chronic pain is a condition in which pain progresses from an acute to chronic state and persists beyond the healing process. Chronic pain impairs function and decreases patients’ quality of life. In recent years, efforts have been made to deepen our understanding of chronic pain and to develop better treatments to alleviate chronic pain. In this review, we summarize the results of previous studies, focusing on the mechanisms underlying chronic pain development and the identification of neural areas related to chronic pain. We review the association between chronic pain and negative affective states. Further, we describe the structural and functional changes in brain structures that accompany the chronification of pain and discuss various neurotransmitter families involved. Our review aims to provide guidance for the development of future therapeutic approaches that could be used in the management of chronic pain.

## 1. Introduction

Pain is a serious and common global medical problem that can cause long-term disability [1,2]. The perception of pain is underpinned by the transduction of mechanical, thermal, and chemical sensory inputs into the subjective awareness of pain [3]. Chronic pain is a condition in which pain progresses from an acute to chronic state, persisting beyond the healing process [4]. As the experience of chronic pain is associated with activity in multiple networks in the central nervous system (CNS), chronic pain is considered a CNS disorder [5]. As a result of multi-network activation in the CNS, chronic pain comprises multiple components, including sensory, emotional, cognitive, and behavioral elements [6]. 

Patients who suffer from painful conditions may demonstrate various clinical outcomes. In most cases, pain-inducing conditions resolve over time, as the body goes through the normal healing process. In a subset of cases, pain progresses into a chronic condition in which pain persists for many years [3]. In recent years, numerous clinical and animal studies have investigated the mechanisms underlying chronic pain to elucidate its cellular and molecular mechanisms. Understanding the mechanisms of development of chronic pain will guide the search for novel therapeutic options for chronic pain. The primary goal of this review is to explore the mechanisms of development of chronic pain. We review the current knowledge of neural areas related to chronic pain, the relationship between chronic pain and negative affective states, structural and functional changes that occur in brain structures during the development of chronic pain, and various neurotransmitter systems involved in the chronification of pain.

## 2. Pain Pathways and Mechanisms of Chronic Pain 

Pain can be classified into three major types: Nociceptive, inflammatory, and neuropathic pain. Nociceptive pain is the response of sensory systems to actual or potentially harmful stimuli detected by nociceptors around the body. Inflammatory pain is associated with tissue damage and the resulting inflammatory process, which may lead to responses such as hyperalgesia, allodynia, and sympathetically maintained pain [7]. Neuropathic pain is a localized sensation of unpleasant discomfort caused by damage or disease in the peripheral and/or central nervous system that persists after a primary lesion or dysfunction. Neuropathic pain may be associated with allodynia, which refers to pain sensitization to stimuli that do not normally provoke pain (non-painful stimulation) [7]. Hyperalgesia refers to an abnormally increased sensitivity to pain, which is associated with hypersensitivity to stimuli. Primary hyperalgesia occurs directly in damaged tissues, whereas secondary hyperalgesia occurs in areas surrounding damaged tissues due to pain-related mediators binding to receptors around the injury site, causing sensitization of adjacent uninjured tissues to mechanical stimuli [7]. 

**Figure 1:**
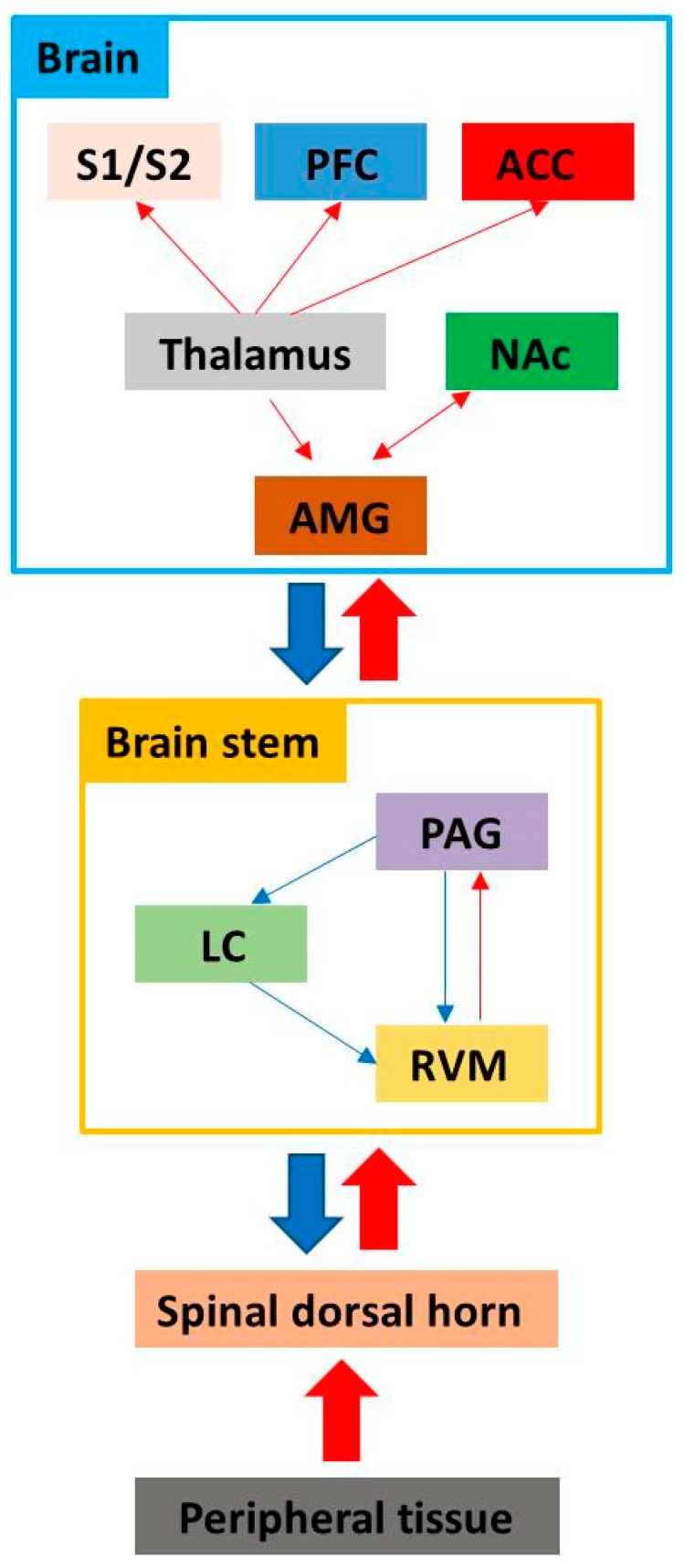
Ascending pathway (red line): A nerve pathway that projects upwards from the spinal cord to the brain carrying sensory information from the body to the brain. Pain signals ascend from the spinal dorsal horn to the rostral ventral medulla (RVM) and periaqueductal grey matter (PAG). Pain signals are then transmitted to the thalamus, where they are sent to higher brain centers, such as the primary and secondary somatosensory cortices (S1/S2), prefrontal cortex (PFC), anterior cortex (ACC), amygdala (AMG), and nucleus accumbens (NAc). Descending pathway (blue line): A nerve pathway that descends down the spinal cord and has a role in the modulation of pain, involving important areas of the brainstem such as the RVM, PAG, and locus coeruleus (LC).

Pain pathways comprise a complex sensory system, which is activated to provide protective responses to noxious stimuli. Input regarding noxious stimuli is transmitted from nociceptors by primary afferent Aδ and C fibers. These fibers have cell bodies located in the dorsal root ganglion and synapse with neurons in the spinal dorsal horn. Various neurotransmitters such as glutamate, calcitonin gene-related peptide (CGRP), and substance P are released as part of signal transduction [8,9,10,11]. Primary nociceptive afferents synapse onto neurons in the Rexed laminae I and II and make connections with neurons located deeper in the dorsal horn, which play a role in signaling the presence, location, and intensity of pain [12]. Projection neurons from the dorsal horn decussate at the ventral commissure and ascend in the lateral spinothalamic tract to the ventral posterolateral nuclei of the thalamus. Finally, the information is transmitted to the somatosensory cortex and periaqueductal gray matter (PAG) [9,13]. Nociceptive information is transmitted to brain areas involved in memory and affective aspects of pain, such as the amygdala, hypothalamus, PAG, and nucleus accumbens (NAc) through the spinoreticular and spinomesencephalic tracts [9,14]. These brain regions, including the somatosensory cortex, PAG, amygdala, hypothalamus, and NAc, are associated with supraspinal responses of pain pathways [15,16,17,18]. Descending pain modulatory systems involve the PAG and rostral ventral medulla (RVM). The RVM is the major output node in the descending modulation of nociception. It receives input from the PAG and sends diffuse bilateral projections to the dorsal horn, terminating at multiple levels [19]. The ascending and descending pain pathways are summarized in Figure 1.

Peripheral injury activates both neuronal and glial cells, and subsequent neuron-glia interactions influence pain hypersensitivity [20]. Glia are nonneuronal cells that support and protect neurons in both the central and peripheral nervous systems. Glia comprise microglia, astrocytes, oligodendroctyes, and radial cells [21]. The development of pain is affected by glial cells, which release neurotransmitters and molecules involved in pain pathways. Glial cells initiate a series of signaling cascades that regulate pain processing at the spinal and supraspinal levels. Glial cells also release inflammatory cytokines and chemokines which may facilitate pain transmission through coupling to neuronal glutamate receptors. Bidirectional neuron–glia interactions affect processing, expression, and transmission of pain and play a critical role in the development and maintenance of pain [21].

Central sensitization is triggered by increased nociceptive input caused by injury or inflammation and is an outcome of physiological plasticity and long-lasting changes in the CNS [22]. Increases in primary afferent fiber responses, as well as increased spontaneous activity and excitability of dorsal horn neurons and receptive field areas, are associated with central sensitization [22,23]. The mechanism of central sensitization involves glutamate signaling via postsynaptic N-methyl-D-aspartate (NMDA) receptors. Activation of NMDA receptors results in the opening of ion channels and calcium influx. This calcium flow plays a critical role in synaptic plasticity in both excitatory and inhibitory synapses. Synaptic plasticity can sensitize the central nociceptive system, resulting in pain hypersensitivity and persistent pain [24]. 

## 3. Pain and Negative Affective States

Brain structures, including the primary somatosensory cortex, secondary somatosensory cortex, anterior cingulate cortex (ACC), prefrontal cortex (PFC), insular cortex, amygdala, thalamus, cerebellum, and PAG, have been identified as regions associated with the perception of pain [15,16,17,18]. The ventral tegmental area (VTA) and NAc, structures comprising the mesolimbic reward circuit, are involved in chronic pain. The prefrontal region and limbic system (ACC, amygdala, VTA, and NAc) are associated with affective aspects of pain and regulate emotional and motivational responses [16,17,25]. These brain regions are not activated separately; they are functionally connected and contribute in a combined fashion to pain processing. Changes in emotional and motivational cues can affect the intensity and degree of pain experience [26]. 

Several studies have investigated the brain areas associated with emotional aspects of pain. Baliki et al. showed that patients with persistent back pain had greater functional connectivity between the medial PFC (mPFC) and NAc [27]. Since the mPFC and NAc are involved in emotion, motivation, and reward-related behaviors, this suggests that the processing of pain perception can be influenced by changes in these functional connections. Hashmi et al. also suggested that regions involved in processing emotions, such as the mPFC and amygdala, are associated with the chronification of pain [28]. Changes in emotion, motivation, and reward-related circuits of the brain (which encode emotional features of pain) may cause disorders associated with emotion in chronic pain conditions. 

A number of neuroimaging studies have demonstrated that morphological changes in corticolimbic structures and emotional systems are associated with persistent pain. Patients with persistent subacute back pain show a reduction in gray matter density in the insular cortex, primary somatosensory cortex, motor cortex, and NAc [27]. The transition of brain activity from the insular cortex, ACC, thalamus, and basal ganglia to the mPFC and amygdala (structures associated with emotion-related circuitry) manifests in patients with persistent subacute back pain [28]. Furthermore, patients with chronic pain conditions show reductions in gray matter volume in the hippocampus and amygdala. Given the functions of these two regions, this reduction suggests that chronic pain may be correlated with emotional and cognitive changes [29,30]. In summary, functional and structural changes in the corticolimbic system and corticolimbic interactions in patients with chronic pain can contribute to emotional and cognitive problems [31]. 

To date, several studies have demonstrated the high comorbidity of affective disorders in patients with chronic pain. Many patients with chronic pain also have severe depression. Patients with chronic pain-induced depression have poorer prognosis than those with chronic pain alone [32]. Chronic pain and depression share similar changes in neuroplasticity and involve overlapping neurobiological mechanisms; monoamine neurotransmitters such as serotonin, dopamine, and norepinephrine are decreased in both chronic pain and depression patients [33,34]. Additionally, brain regions involved in pain pathways, such as the prefrontal cortex, hippocampus, and amygdala, are similar to those involved in mood disorders [33,35]. 

As pain develops into a chronic condition, negative emotional states may be accompanied by other emotional disorders such as anxiety, anhedonia, cognitive deficits, sleep disturbances, and suicide [15,36]. The prevalence of suicidal ideation and suicide attempts is noticeably higher in patients with chronic pain than in control patients [37]. A recent review indicated that chronic pain itself is an important independent risk factor for suicidality regardless of type and concluded that depressive symptoms, anger problems, and harmful habits are general risk factors for suicidality in patients with chronic pain [38]. 

Several animal studies have demonstrated the negative affective disorders associated with pain. A study using a rat model with a chronic constriction injury reported that long-term pain led to an anxiety-like profile, increased responses to aversion, and impairments in cognitive tasks [39]. Wu et al. reported that negative effects including pain aversion and anxiety were associated with hyperalgesia but that the manifestations of negative effects may occur over different time courses, suggesting that therapy should be targeted based on the different stages of pain and its comorbidities [40]. 

Chronic pain and various affective disorders are often managed poorly [41,42]. Understanding the affective aspects related to chronic pain may facilitate the development of novel therapies for more effective management. 

## 4. Pain and Long-Term Functional Changes in Corticolimbic Structures 

The corticolimbic system is a mediator of chronic pain and plays an important role in the development, maintenance, and amplification of chronic pain [3,43]. Pain chronification is accompanied by spatiotemporal reorganization of brain activity, with a transition from sensory regions to emotional and limbic regions of the brain [13]. Structural and functional plasticity in the corticolimbic circuitry accompanies the transition from acute to chronic pain. When nociceptive signals persist, the corticolimbic circuitry stays activated. Through the interactions with the prefrontal cortical circuitry, nociceptive state progresses to a more emotional state. The persistent activation of the corticolimbic circuitry brings functional and anatomic alterations to the cortex, resulting in pain chronification [44]. Corticolimbic structures that are associated with pain and their long-term functional changes are described below (Table 1). 

### 4.1. Prefrontal Cortex

The mPFC is an important region for top-down cognitive control over emotion-driven behaviors [31]. The mPFC is a critical region involved in emotional and cognitive processing in chronic pain [45]. The prelimbic and infralimbic mPFCs receive inputs from brain regions including the basolateral amygdala (BLA), hippocampus, thalamus, and contralateral mPFC and send excitatory projections to the amygdala [31]. Chronic pain is considered to develop as a result of the persistence of pain memory and inability to erase pain memory after injury [43]. Considering its importance in extinction of fear behaviors, impaired mPFC activation could lead to a failure in the elimination of subcortically driven fear behaviors, thereby resulting in pain chronification [31]. Preclinical evidence suggests that mPFC function is associated with pain states, based on electrophysiology studies in anesthetized rats showing a reduction in evoked and background activity in the mPFC in acute arthritis pain [46,47]. A decrease in mPFC volume has also been observed in patients with chronic pain [45]. 

### 4.2. Anterior Cingulate Cortex

The ACC is associated with affective and motivational aspects of pain [48,49,50,51]. The ACC is involved in the processing and modulation of pain. Nociceptive inputs are sent from the medial thalamus to ACC and combined with motivation and affective information received from other areas of the brain, such as the insular cortex, mPFC, and BLA [16,49,52,53,54]. The ACC then generates affective and motivational pain responses through its projections to the amygdala, NAc, and mPFC [49,52,53,54,55]. Additionally, the interactions of the ACC with pain neurocircuitry in the PAG have been reported, which accounts for the activation of the ACC and PAG in the presence of noxious stimuli [16]. The activation of ACC-PFC-PAG circuity and increased activity in the ACC is associated with negative emotions [16,56]. 

### 4.3. Amygdala

The amygdala is associated with emotions and affective disorders [50,51,57]. Studies have reported activation of the amygdala in pain states, suggesting that the amygdala plays an important role in emotional affective aspects of pain [3,50,51,52,58,59]. The amygdala receives cortical and thalamic inputs, and the lateral/basolateral (LA/BLA) complex of the amygdala adds emotional and affective context to sensory information [50,51,52]. This information is then sent to the central nucleus of the amygdala, which comprises γ-aminobutyric aid (GABA)-ergic neurons and regulates fear and pain [50,51,52]. Preclinical studies have demonstrated that neuronal excitability is increased in the central nucleus of the amygdala in neuropathic pain [60,61]. 

### 4.4. Hippocampus

The hippocampus is part of the limbic system, which plays an important role in declarative and episodic memory [62]. The hippocampus possesses extensive nerve fiber connectivity with other brain regions involved in emotion and cognition [31,63]. It regulates the hypothalamic–pituitary–adrenal axis, which makes it vulnerable to neuropsychiatric disorders such as stress and depression [63]. Volumetric changes in the hippocampus are associated with increased risk of depressive disorders, and decreased hippocampal volumes in patients with depression have been reported [63,64]. 

Changes in the hippocampus have been reported in chronic pain conditions. Hippocampal neurogenesis contributes to learning and memory and may trigger the development of chronic pain. The upregulation of hippocampal neurogenesis resulted in the prolongation of persistent pain [65]. Chronic pain is generally accompanied by cognitive deficits and aversive emotional states, including depression and anxiety disorders. Functional and structural changes in the hippocampus, such as decreased hippocampal neurogenesis, are closely associated with memory deficits and aversive affective states in patients with chronic pain [31,65]. 

### 4.5. Nucleus Accumbens

The NAc is a forebrain structure that integrates cortical and affective information and assigns motivation and value for the selection of appropriate behavioral responses [43,54,55,66,67,68,69]. The NAc participates in emotional learning, evaluation of reward signals, and encoding of salience for pain [70]. Changes in NAc circuitry and connectivity are risk factors for pain chronification. A brain-imaging study reported that changes in NAc circuitry were predictive of the transition to chronicity in patients with back pain [71,72]. The transition to chronic pain is influenced by NAc plasticity. 

### 4.6. Periaqueductal Gray Matter

The PAG is located in the brain stem and is divided into three subregions: ventrolateral, lateral, and dorsolateral [73]. The PAG plays an important role in both the ascending and descending modulation of nociception and regulates other autonomic and emotional behaviors [74]. It projects to the rostroventral medulla, which sends descending inhibitory and excitatory fibers to the dorsal horn of the spinal cord [75]. The PAG integrates information received from higher centers of the brain and receives ascending nociceptive input from the dorsal horn. The PAG regulates the processing of nociceptive information in the dorsal horn of the spinal cord and plays a critical role in the descending modulation of pain [7]. 

## 5. The Role of Neurotransmitters in Chronic Pain 

Neurotransmitters are chemical substances that mediate transmission of impulses across the synapse. The transmission of neuronal signals across the synapse is initiated with the release of neurotransmitters from the presynaptic neuron. Neurotransmitters are released into the synaptic cleft and bind to neurotransmitter receptors on postsynaptic neurons. Neurotransmitters can be classified based on their function (excitatory or inhibitory), molecular size (small molecules, including amino acids and monoamines, or large molecules, including peptides), or type (inflammatory mediators, including prostaglandin E2, adenosine triphosphate, adenosine, histamine, glutamate, and nitric oxide (NO), or non-inflammatory mediators, including GABA, CGRP, peptides, glycine, and cannabinoids) [7]. The binding of neurotransmitters to their receptors on postsynaptic neurons influences pain transmission in either an inhibitory or excitatory way. Glial cells, such as microglia and astrocytes, release various neurotransmitters that contribute to the development and maintenance of chronic pain by activating or deactivating nociceptive neurons in the CNS [76,77,78]. 

### 5.1. Neuropeptides

Neuropeptides are a family of neurotransmitters that are relatively large molecules from a structural perspective. Neuropeptides constitute a diverse group of signaling molecules that are widely distributed in primary sensory neurons in the dorsal root ganglion of the spinal cord and also in the brain. Neuropeptides play various roles in the formation, transmission, modulation, and perception of different types of pain. Neuropeptides that affect pain sensitivity by modifying the activity of glial cells have been recently reported, including proopiomelanocortin (POMC)-derived peptides, neuropeptide Y, vasoactive intestinal peptide (VIP), pituitary adenylate cyclase-activating polypeptide (PACAP), somatostatin, cortistatin, tachykinins, CGRP, adrenomedullin, and ghrelin [77,79,80]. These neuropeptides bind to specific receptors expressed on microglial cells and influence pain processes, including neuroinflammation, neurodegeneration, and neuromodulation. 

### 5.2. Glutamate

Glutamate is an excitatory neurotransmitter which plays an important role in neuronal activation. Glutamate mediates synaptic transmission of sensations such as pain and itchiness. Glutamate also participates in the generation of long-term plastic changes in the cortex and is a key player in central sensitization [11,81]. Glutamate receptors are involved in neurotransmission and plasticity at the level of the spinal cord. Once pain signals are transmitted, glutamate plays a critical role in the storage and generation of long-term memories [82]. Glutamate receptors comprise ionotropic and metabotropic subtypes. Alterations in these receptors increase synaptic strength and neuronal excitability [83]. Ionotropic glutamate receptors form an ion channel pore, which is activated when glutamate binds to the receptor, whereas metabotropic glutamate receptors indirectly activate ion channels through signaling cascades involving G proteins [81]. 

The ionotropic glutamate receptors include N-methyl-d-aspartate (NMDA), α-amino-3-hydroxy-5-methyl-r-isoxazoleproprionic acid (AMPA), and kainite (KA) receptors. NMDA receptors contribute to the initiation of long-term plasticity such as long-term potentiation [84], while AMPA receptors are involved in synaptic transmission and plasticity [85]. NMDA and AMPA receptors are both involved in the development of chronic pain and depression [33,86,87]. KA receptors are involved in sensory transmission at higher intensities and regulate synaptic transmission presynaptically [81]. Increased activity in the excitatory system and attenuation of the inhibitory system by glutamate induce central hyperalgesia, leading to persistent pain. 

### 5.3. Gamma-Aminobutyric Acid (GABA)

GABA is an inhibitory neurotransmitter in the CNS that reduces neuronal excitability and regulates muscle tone. GABA is involved in pain modulation by regulating the transmission of nociceptive signals through the activation of GABA receptors located on primary afferent terminals and in the dorsal horn [88]. Stimulation of GABA receptors leads to that inhibition of voltage-gated calcium channel activity and inhibits the release of other neurotransmitters, such as glutamate, substance P, and CGRP [89]. In neuropathic and chronic inflammatory pain conditions, GABAergic inhibitory control is decreased, leading to increased excitation and central sensitization [82]. 

### 5.4. Neurotrophic Factors

Neurotrophic factors are molecules that regulate the growth of neurons and contribute to the proliferation of nociceptive axons and terminals in peripheral tissues, which may result in persistent pain conditions [90]. Among these factors, nerve growth factors (NGFs), a neurotrophin subfamily of growth factors, play a role in the development and modulation of persistent pain [91,92]. NGF is a neurotrophic factor or neuropeptide which is released locally by fibroblasts at the site of injury [7]. The binding of NGF to receptors on peripheral nociceptors triggers rapid sensitization of the nociceptive response [10,93]. NGF signaling also induces pain by promoting the sprouting of nociceptive fibers in peripheral tissues, which causes hyperinnervation [91]. Preclinical studies have reported that anti-NGF treatments can reduce markers of peripheral and central sensitization, can reverse hyper-innervation of peripheral tissues, and can mitigate pain-related behaviors [94,95,96]. 

Interleukin-6 (IL-6), another neurotrophic factor, is an important mediator in pain processing [91]. Following nerve injury, IL-6 is released and its levels are increased. IL-6 is involved in the development of pain and CNS sensitization [97,98]. It promotes and mediates various inflammatory pain conditions [99]. 

Inhibition of neurotrophic factors with the goal of developing medications to better manage chronic pain is an active area of investigation. Potential harmful effects of neurotrophic factor inhibitors in the treatment of chronic pain warrant further research. 

### 5.5. Nitric Oxide

NO is a freely diffusible, soluble gas with a half-life of seconds [100]. NO is synthesized by nitric oxide synthase (NOS) and is primarily regulated by the expression and activity of NOS [100]. There are three different NOS isoforms: neuronal NOS (nNOS), inducible NOS (iNOS), and endothelial NOS (eNOS) [101]. Among these, nNOS is the major source of NO involved in pain processing, which occurs in the dorsal horn of the spinal cord. The number and density of nNOS in dorsal horn neurons are increased during the inflammatory process of pain induced by proinflammatory agents [101,102,103]. During neuropathic pain, nNOS expression is upregulated in the dorsal root ganglion (DRG) which leads to an increase in the number of nNOS-positive DRG neurons [101,104]. Therefore, nNOS seems to be involved in the processing of persistent inflammatory and neuropathic pain in the spinal cord. NO induces cyclic guanosine monophosphate formation in cells of the nociceptive system and activates downstream targets. While evidence suggests that the production of NO can reduce pain through its antinociceptive effects, the inhibition of NO can also reduce inflammatory and neuropathic pain [101]. Focusing on specific NO-dependent signaling mechanisms is necessary when targeting NO for the treatment of pain. 

### 5.6. Opioid Peptides

Opioid peptides are a family of neuropeptides that bind to opioid receptors, including μ, δ, and κ receptor subtypes [105]. Opioid receptors are abundantly distributed in both primary afferent neurons and dendrites of postsynaptic neurons. Enkephalin and dynorphin are two endogenous opioid peptides that inhibit the release of excitatory neurotransmitters from afferent terminals and reduce neuronal excitability, resulting in decreased pain sensation [7]. Changes in the capacity of brain regions to respond to endogenous or exogenous opioids are related to decreased opioid receptor expression, which may underscore the lack of efficacy of opioids in chronic pain. Reduced opioid receptor availability, reflecting decreased receptor expression, contributes to the development of chronic pain [106].

### 5.7. Endocannabinoids

Cannabinoids are a class of neurotransmitters present in pain signal transduction pathways. After injury, neural and nonneural cells release arachidonic acid derivatives known as endocannabinoids [107]. Endocannabinoids regulate neural conduction of pain signals by attenuating sensitization and inflammation via the activation of cannabinoid receptors type 1 and type 2 (CB1 and CB2), which are located at peripheral, spinal, or supraspinal sites [108]. CB receptors modulate neuroimmune interactions and inflammatory hyperalgesia. Cannabinoids inhibit the release of presynaptic neurotransmitters and neuropeptides, modulate postsynaptic neuronal excitability, activate descending inhibitory pathways, and reduce neuro-inflammatory signaling [109,110]. 

Cannabis has been used throughout the world for centuries, both legally and illegally [111]. Although cannabis is usually not the first drug of choice for patients with acute pain, several studies have reported that the use of cannabinoids is significantly associated with a reduction in pain and may be an effective approach for pain management [112,113]. Long-term studies evaluating treatment effects of exogenous cannabinoids should take into consideration the efficacy, therapeutic window, dose-dependent effects, and side effects to provide support for the use of cannabinoids in pain treatment [109]. 

### 5.8. Leptin and Orexin 

Leptin is a peptide produced by adipose tissue. The concentration of leptin is regulated by nutritional state. Leptin expression is increased following food intake and suppresses appetite, whereas leptin expression is decreased with fasting. Although leptin is known to be involved in the modulation of pain signals, its role in the pathogenesis of neuropathic pain is controversial. Studies using a rat model reported that leptin induced interleukin-1β production and enhanced production of pronociceptive mediators such as cyclooxygenase-2, iNOS, and matrix metalloprotrease-9, linking adipokines to the development of neuropathic pain [114,115]. However, another study reported that acute leptin administration after injury prevented the development of thermal hyperalgesia and mechanical allodynia and reduced inflammatory mediators [116]. The role of leptin should be clarified in future studies. 

In addition, orexin-A and B (also known as hypocretin-1 and-2) are neuropeptides involved in the modulation of nociception. Orexin signaling in some spinal and supra-spinal sites regulates descending pain modulatory circuitry. The involvement of the orexinergic system in pain modulation suggests that orexins may be potential therapeutic targets for chronic pain treatment [117].

### 5.9. Melatonin 

Melatonin is a hormone produced from tryptophan in the pineal gland. It is involved in the control of circadian rhythms and associated physiological responses such as sleep, anxiety, and pain [118]. The development of chronic pain syndromes is associated with the desynchronization of circadian and biological rhythms. The mechanisms underlying the analgesic effects of melatonin include the involvement of β-endorphins, GABA receptors, opioid 1-receptors, and the NO-arginine pathway [119]. Melatonin promotes anti-analgesic effects by interacting with opioidergic, benzodiazepinergic, muscarinic, nicotinic, serotonergic, and α1 and α2-adrenergic receptors [120]. Treatment with melatonin may improve conditions such as migraine, fibromyalgia, irritable bowel syndrome, preoperative anxiety, and postoperative pain [118]. 

## 6. Treatment of Chronic Pain

The prevention and treatment of chronic pain may be achievable with pharmacological and/or psychological interventions. Despite the progress made in understanding how chronic pain occurs, the development of effective drugs capable of controlling chronic pain remains a challenge. Pharmacological agents commonly used in primary care settings include gabapentin and pregabalin, which decrease calcium-mediated release of glutamate and other neurotransmitters. NMDA receptor antagonists (such as dextromethorphan and ketamine) and antidepressants (such as serotonin and norepinephrine reuptake inhibitors and tricyclic antidepressants) are also effective in the management of chronic pain conditions [13]. Other pharmacological interventions targeting a variety of ion channels, neurotransmitters, and receptors are under investigation for prevention or reversal of the reorganization of chronic pain pathways [13,121,122]. 

Psychological interventions for chronic pain include operant behavioral therapy, cognitive behavioral therapy, and motivational interviewing [123,124]. These approaches focus on factors that influence chronic pain, such as failure to obliterate pain responses and maladaptive learned responses. Other approaches include strategies related to maladaptive brain plasticity, such as imagery, mirror training, and virtual reality. These novel treatments aim to reverse the distorted sensory input and motor output, and to restore correct information by enhancing a coherent body image in patients with chronic pain [13,125]. 

Repetitive transcranial magnetic stimulation (rTMS) is a noninvasive brain stimulation technique used to reduce chronic pain by directly altering brain activity by electrical stimulation [13]. rTMS targeting the primary motor cortex reduces pain by triggering activity in descending inhibitory pathways to the dorsal horn. Additionally, rTMS is thought to exert antinociceptive effects by altering neuronal activity in the PAG matter related to pain processing. rTMS also influences the endogenous opioid system, which can control chronic pain [126,127]. 

Integrative strategies such as exercise, yoga, and nutrition have been suggested to have analgesic effects [128]. During and after exercise, different endogenous systems are activated and various neurotransmitters that modulate pain perception are released, resulting in increased levels of endogenous opioids, nitric oxide, serotonin, anti-inflammatory cytokines, brain-derived neurotrophic factor, and endocannabinoids [129]. The practice of yoga postures increases brain GABA levels, improves mood, and decreases anxiety [130]. Nutrition is also important for the treatment of painful and inflammatory conditions. Anti-inflammatory diets can prevent many chronic diseases that are associated with pain; nutritional interventions such as gluten- or grain-free diets or the elimination of nightshade vegetables from the diet may help to alleviate chronic pain conditions [131]. 

For effective treatment of chronic pain, various therapeutic approaches described above should be used in combination. However, despite the application of several therapeutic methods, some patients will experience persistent pain, which affects quality of life. To address this persistent need, efforts to identify novel therapeutic targets should be continued. Additionally, further research on the modulation of synaptic plasticity and the identification of novel neurotransmitter targets for the management of refractory chronic pain are warranted.

## 7. Discussion 

Chronic pain is a critical medical problem worldwide, characterized by a high prevalence and significant cost. If pain becomes chronic, it can significantly reduce quality of life and cause depression, suicide, insomnia, impaired cognitive function, and other deleterious effects [15,32,36]. In order to develop appropriate therapeutic targets for chronic pain, it is important to understand factors that affect the transition of acute pain to chronic pain and the mechanisms underlying the development of chronic pain. In addition, the reasons underpinning the high comorbidity between chronic pain and negative affective states should be investigated to identify appropriate treatments.

This review provides an overview of existing knowledge on the mechanisms underlying the development of chronic pain and neural areas related to chronic pain. The development of chronic pain is associated with synaptic plasticity and changes in the CNS and various neural areas that modulate pain. Chronic pain entails structural and functional changes in corticolimbic brain regions such as the prefrontal cortex, ACC, amygdala, hippocampus, NAc, and PAC. Changes related to chronic pain can induce negative affective states such as depression, anger, and anxiety, underpinned by common neuroplasticity changes in chronic pain and negative affective states [32,38]. It is important to note that the transmission of pain signals across the synapse involves various neurotransmitters released by glial cells, such as neuropeptides, glutamate, GABA, and neurotrophic factors, which play an important role in the development of chronic pain. 

We acknowledge that we have not addressed all the factors influencing the shift from acute to chronic pain and changes in areas related to pain. Pain has several important dimensions, including sensory, emotional, and cognitive dimensions. The sensory dimension of pain involves how we perceive pain signals and the amount of pain we recognize. The emotional dimension of pain indicates how we feel about experiencing pain. The cognitive dimension of pain entails how we interpret pain and how we respond to pain stimuli. In the future, correlations or interactions among these dimensions of pain should be elucidated. Future studies should investigate factors that trigger pain chronification to provide insight into how acute pain becomes chronic. 

## 8. Conclusions

Chronic pain is one of the most intractable clinical problems faced by clinicians and can be physically and emotionally debilitating. Combinations of treatments are currently used for treating chronic pain, but a subset of patients experience persistent non-endurable pain. Therefore, deeper understanding of the mechanisms and key factors involved in pain chronification is necessary to identify novel therapeutic targets for developing better treatments for chronic pain. 

## Figures and Tables

**Table 1:** Corticolimbic structures associated with chronic pain.

Brain Structures	Location	Function
Medial prefrontal cortex	Located in the frontal lobe	Decision making, self-control, regulation of emotion, processing of risk and fear, and regulation of amygdala activity
Amygdala	Located in the frontal portion of the temporal lobe, close to the hippocampus	Memory modulation, decision-making, reward, and emotional responses
Periaqeuductal gray	Located around the cerebral aqueduct within the tegmentum of the midbrain	Autonomic function, motivated behavior, behavioral responses to threatening stimuli, and primary control center for descending pain modulation
Anterior cingulate cortex	Located in the frontal part of the cingulate cortex	Autonomic functions, attention allocation, reward anticipation, decision-making, ethics and morality, impulse control, emotion, and registration of physical pain
Hippocampus	Located in the medial temporal lobe	Consolidation of memories, emotion, navigation, spatial orientation, and learning
Nucleus accumbens	Located in the basal forebrain	Cognitive processing of motivation, aversion, reward, reinforcement learning, and significant role in addiction

## Abbreviations

CNS	central nervous system
CGRP	calcitonin gene-related peptide
PAG	periaqueductal gray matter
NAc	nucleus accumbens
RVM	rostral ventromedial medulla
NMDA	N-methyl-D-aspartate
ACC	anterior cingulate cortex
PFC	prefrontal cortex
VTA	ventral tegmental area
mPFC	medial prefrontal cortex
LA/BLA	lateral/basolateral complex of the amygdala
GABA	gamma-aminobutyric aid
NO	nitric oxide
POMC	proopiomelanocortin
VIP	vasoactive intestinal peptide
PACAP	pituitary adenylate cyclase-activating polypeptide
AMPA	α-amino-3-hydroxy-5-methyl-r-isoxazoleproprionic acid
KA	kainite
NGF	nerve growth factor
IL-6	interleukin-6
NOS	nitric oxide synthase
nNOS	neuronal nitric oxide synthase
iNOS	inducible nitric oxide synthase
eNOS	endothelial nitric oxide synthase
CB1/CB2	cannabinoid receptors type 1 and type 2
rTMS	repetitive transcranial magnetic stimulation
